# Reduced Hippocampal Volume in Healthy Young ApoE4 Carriers: An MRI Study

**DOI:** 10.1371/journal.pone.0048895

**Published:** 2012-11-09

**Authors:** Laurence O'Dwyer, Franck Lamberton, Silke Matura, Colby Tanner, Monika Scheibe, Julia Miller, Dan Rujescu, David Prvulovic, Harald Hampel

**Affiliations:** 1 Department of Psychiatry, Psychosomatic Medicine and Psychotherapy, Goethe University, Frankfurt, Germany; 2 UMS 3408, CNRS, University of Caen, GIP CYCERON, Caen, France; 3 Department of Zoology, Trinity College Dublin, Dublin, Ireland; 4 Department of Psychiatry, Ludwig-Maximilians-University, Munich, Germany; University of Cambridge, United Kingdom

## Abstract

The E4 allele of the ApoE gene has consistently been shown to be related to an increased risk of Alzheimer's disease (AD). The E4 allele is also associated with functional and structural grey matter (GM) changes in healthy young, middle-aged and older subjects. Here, we assess volumes of deep grey matter structures of 22 healthy younger ApoE4 carriers and 22 non-carriers (20–38 years). Volumes of the nucleus accumbens, amygdala, caudate nucleus, hippocampus, pallidum, putamen, thalamus and brain stem were calculated by FMRIB's Integrated Registration and Segmentation Tool (FIRST) algorithm. A significant drop in volume was found in the right hippocampus of ApoE4 carriers (ApoE4+) relative to non-carriers (ApoE4−), while there was a borderline significant decrease in the volume of the left hippocampus of ApoE4 carriers. The volumes of no other structures were found to be significantly affected by genotype. Atrophy has been found to be a sensitive marker of neurodegenerative changes, and our results show that within a healthy young population, the presence of the ApoE4+ carrier gene leads to volume reduction in a structure that is vitally important for memory formation. Our results suggest that the hippocampus may be particularly vulnerable to further degeneration in ApoE4 carriers as they enter middle and old age. Although volume reductions were noted bilaterally in the hippocampus, atrophy was more pronounced in the right hippocampus. This finding relates to previous work which has noted a compensatory increase in right hemisphere activity in ApoE4 carriers in response to preclinical declines in memory function. Possession of the ApoE4 allele may lead to greater predilection for right hemisphere atrophy even in healthy young subjects in their twenties.

## Introduction

Apolipoprotein E (ApoE) plays a key role in neuronal development with signalling through ApoE receptors and proteins mediating processes including synaptic plasticity, neuronal survival and neurite outgrowth [1], [2]. ApoE also plays an important role in lipolysis [3] and the regulation of lipid transport [4]. There are three allelic variants of the ApoE gene in humans (E2, E3, E4) [5] with the E4 allele consistently being shown to confer a higher risk of developing both early and late onset Alzheimer's disease (AD) [6], [7]. Brain structure and function have been found to be altered in ApoE4 carriers, both in AD patients [8], [9] and in healthy subjects [10]–[14]. Studies have found greater rates of temporal lobe atrophy in AD patients with greater load of E4 allele [8], [9], [15], [16] as well as reduced medial temporal lobe volumes in healthy ApoE4 carriers across the age spectrum [14], [17]–[20]. However, a number of studies have also failed to replicate these findings [21]–[23]. Functional studies have reported both increased [7], [17], [24] and decreased [25], [26] task-related BOLD signals in carrier groups relative to non-carriers.

Specifically within younger cohorts some studies suggest that neuronal deficits related to the E4 carrier genotype may lead to greater recruitment of functional activation in order to reach the same level of cognitive performance as E4 non-carriers [27]–[29]. Other studies have failed to find cognitive differences by ApoE genotype in younger subjects [30], while still more studies have found evidence for beneficial effects of the E4 carrier genotype in young people [26], [31]. Potential cognitive benefits of the ApoE4 genotype is linked with the concept of antagonistic pleiotropy whereby E4 carriers are suggested to have cognitive advantages in early life, which is followed by increased risk of cognitive damage and reduced neuronal efficiency only in later life [32], [33].

Much less work has been done in terms of studying how ApoE genotype influences the structure of the healthy young brain. In older subjects, hippocampal volume has been found to decrease progressively from non-demented older subjects to MCI to AD, with the additional caveat that E4 carriers within each group exhibit significantly smaller hippocampal volumes compared to non-carriers [34]. This also relates to earlier work that noted reduced hippocampal volume and cortical thickness in E4 carriers in healthy middle aged and healthy older people [20], [25], [35]. In children and young adolescents, thickness of the entorhinal cortex has also been linked to ApoE4 carrier status [19]. However, not all studies have found hippocampal volume to be reduced in E4 carriers [26].

Interestingly, a meta-analysis of 82 studies found that right hippocampal volume is larger than the left in healthy adults [36]. Decreased hippocampal asymmetry [37] and diminished right hippocampal volume have been noted in healthy elderly subjects that were carriers of the E4 allele [38]. It has also been suggested that changes in “normal” asymmetry may be a potential indicator of early pathology [37], [39]–[41].

The aim of the current study was to investigate the effect of APOE genotype on deep grey matter (GM) structures in healthy young people. FMRIB's Integrated Registration and Segmentation Tool (FIRST) [42] was applied to detect significant differences that may be present between the groups. To the best of our knowledge, no work to date has quantified volumetric differences in deep GM structures between healthy young E4 carriers and non-carriers. Here FIRST is used to segment 15 deep GM structures in a semi-automated manner. The primary research question that was addressed was whether or not ApoE genotype affects the volume of deep GM structures in healthy young people.

## Methods

### Ethics Statement

The study was approved by the Ethics Committee of Goethe University and was in accordance with the Declaration of Helsinki. All participants provided informed written consent.

### Participants

44 cognitively intact persons between 20 and 38 years of age (mean = 26.8, S.D = 4.6), all without any history of neurological or psychiatric disease were assessed in the current study. These 44 subjects were drawn from a larger cohort of 96 subjects. All of the 44 selected subjects were right-handed, as assessed with the Edinburgh Handedness Inventory [43] and provided written informed consent. Ethics approval was obtained from the local ethics committee of JWG University Frankfurt. All subjects underwent neuropsychological assessment. Verbal learning and memory was assessed using the German Version of the California Verbal Learning Test (CVLT) [44], [45], visual memory was tested with the Brief Visual Memory Test - R (BVMT R) [46]. Additionally, measures of working memory and attention were obtained using the Letter Number Sequencing (LNS) [47], Spatial Span of the Wechsler Memory Scale 3 (WMS SS) [48] and Trail Making Test A (TMT). The verbal IQ was tested with a German verbal intelligence test (Mehrfachwahl-Wortschatz-Test B; MWTB), in which subjects had to indicate real words within lists of pseudo-words [49]. Depressive Symptoms were measured with the German Version of the Beck Depression Inventory (BDI 2) [50], [51].

All participants from the larger cohort (n = 96) underwent APOE genotyping using PCR and sequencing. For the current analysis, 21 subjects who were heterozygote for ApoE4 (ε3/ε4) and one subject who was homozygote for ApoE4 were included into the ε4+ group. 22 subjects, matched for age, gender and education who were ε4 negative (ε3/ε3) were included into the ε4- group. Group characteristics are summarized in Table 1.

**Table 1:** Demographic and cognitive characteristics of the sample groups.

	APOE4 non-carriers		APOE4 carriers			
Variable	Mean	SD	Mean	SD	T-value	P-value
	n = 22			n = 22		
Age (years)	26.73	4.00	26.86	5.28	5.28	0.92
Gender (m/f)	13/9			13/9		0.76
Education (years)	16.83	4.46	17.04	4.34	−0.15	0.88
MWTB	29.71	3.61	30.27	4.31	−0.46	0.65
MWTB IQ	106.70	23.63	114.71	15.34	−1.28	0.21
TMT (sec)	22.00	5.85	19.27	3.94	1.81	0.08
WMS SS	19.14	1.98	19.36	2.82	−0.31	0.76
LNS	18.73	3.22	17.77	2.65	1.07	0.29
BVMT R	32.67	3.47	32.00	3.61	0.62	0.54
BDI 2	3.23	3.58	2.41	2.92	0.83	0.41
CVLT	66.82	7.96	64.50	9.05	0.90	0.37

Values are mean ± standard deviation. Significance was set at p<0.05; thus no significant differences were found between the groups. Values denote mean and standard deviation or number of subjects. P-values refer to t-tests (parametric tests) and chi-square tests (for categorial data). Abbreviations: MWTB: Mehrfachwahl-Wortschatz-Test B, a German Verbal intelligence test; TMT: trail making test; WMS SS: Spatial Span of the Wechsler Memory Scale; LNS: Letter Number Sequencing; BVMT R: Brief Visual Memory Test R; BDI 2: Beck Depression Inventory 2; CVLT: California Verbal Learning Test.

### ApoE4 Genotyping

APOE genotyping of the two determinating variants rs7412 and rs429358 was analyzed using pre-designed TaqMan SNP Genotyping assays (Applied Biosystems, Foster City, CA). Briefly for each SNP 20 µl reaction mix contained 15 ng genomic DNA, unlabeled PCR primers, MGB labeled probes (VIC, 6FAM), 10 µl of 2× TaqMan universal PCR Master Mix (Applied Biosystems, Foster City, CA). PCR was performed on an ABI 7000 instrument (Applied Biosystems, Foster City, CA) with the following cycling programm: 95°C for 15 s, 40 cycles of 95°C for 15 s and 60°C for 60 s. The ABI 7000 genotyping software was used for allelic discrimination.

### Imaging Methods

All MR images were acquired using a Trio 3-T scanner (Siemens, Erlangen, Germany) with a standard head coil for radiofrequency transmission and signal reception. Participants were outfitted with protective earplugs to reduce scanner noise and a hand-held response device. For T1 weighted structural brain imaging, an optimized 3D modified driven equilibrium Fourier transform (3D MDEFT) sequence was used with the following parameters: acquisition matrix = 256×256, repetition time (TR) = 7.92 ms, echo time (TE) = 2.48 ms, field of view = 256 mm, 176 slices, 1.0 mm slice thickness.

A T2-weighted fluid attenuation inversion recovery (FLAIR) sequence was also acquired to ensure that vascular pathology was not significant. For all 44 subjects selected from the larger cohort, no hyperintense white matter lesions were seen in the FLAIR scans.

### High Resolution T1W Structural Image Processing

Images were skull stripped with the Brain Extraction Tool (BET) from the FSL library. Brain tissue volume, normalised for subject head size, was estimated with SIENAX [52], [53], which is part of the FSL library. SIENAX starts by extracting brain and skull images from the single whole-head input data. The brain image is then affine-registered to MNI152 space [54], [55] (using the skull image to determine the registration scaling); this is primarily in order to obtain the volumetric scaling factor, to be used as a normalisation for head size. The scaling factor is derived from the normalisation matrix [53]. Next, tissue-type segmentation with partial volume estimation is carried out [56] in order to calculate total volume of brain tissue including separate estimates of volumes of WM and GM. Both normalised and absolute volumes of WM and GM were obtained.

### FIRST Structural Image Processing

**Figure 1:**
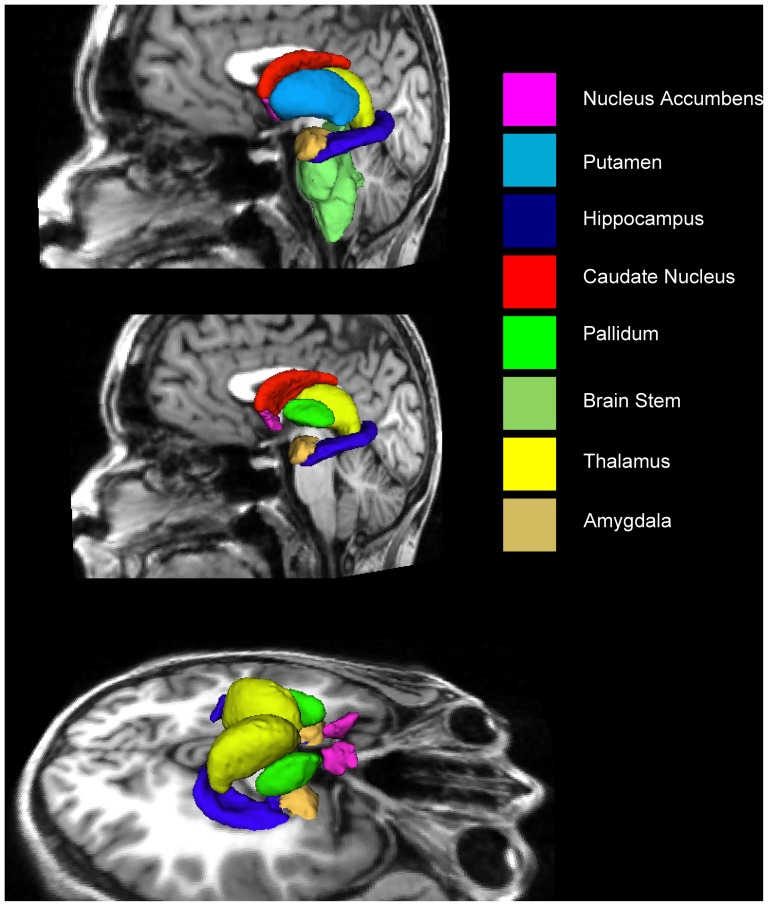
FIRST segmentation of a sample subject. **In the middle panel, the putamen is removed to reveal pallidum (bright green).**

The algorithm FIRST, was applied to separately estimate the left and right volumes of seven subcortical regions; amygdala, hippocampus, nucleus accumbens, caudate nucleus, putamen, pallidum, thalamus and brain stem. FIRST is part of FMRIB's Software Library (FSL) and performs both registration and segmentation of the regions noted above [42]. During registration, the input data (3D T1 images) are transformed to the MNI (Montreal Neurological Institute) 152 standard space, by means of affine transformations based on 12 degrees of freedom. After subcortical registration, a sub-cortical mask is applied, to locate the different subcortical structures, followed by segmentation based on shape models and voxel intensities. Absolute volumes of subcortical structures are calculated, taking into account the transformations made in the first stage [42]. After registration and segmentation of all 44 scans, all segmented subcortical regions were examined visually for problems with registration or segmentation. No errors were found. An example of subcortical segmentation of a representative subject is shown in Figure 1.

To obtain neocortical GM volume (NeoCorGM) independent frorm the deep GM structures of interest, we subtracted the volumes of the hippocampus and amygdala from the absolute GM volume as given by SIENAX. Intracranial volume (ICV) was calculated by adding the volumes of cerebral spinal fluid, total GM and total WM together. Individual differences in brain size were corrected by dividing the volumes of specific deep GM structures by ICV. Thus the following formula was used to compute normalised volumes of each deep grey matter structure:
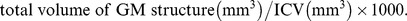



### Statistical Analysis

R statistical software, including the lme4 package [57] was used for all statistical analysis [58]. In the current study, the “glmer” function was used to fit a generalised mixed-effects model using maximum likelihood (ML). Generalised mixed effects models are mixed effects models in which both the fixed and random effects contribute linearly to the response function. Fixed effects influence the mean of the response, while random effects influence the variance of the response. The normalised volumes of deep grey matter structures together with gender were set as fixed effects. Age was included as a random effect. Thus, the variance that arises from differences in age among participants is accounted for in all models. The response variable was set as genotype. The models are thus assessing the influence of genotype on structural grey matter volumes.

Two separate models were investigated: a model for the effect of genotype on the normalised volumes of right hemisphere structures and a model of the effect of genotype on the normalised volumes of the left hemisphere structures. The starting model for the right hemisphere was:
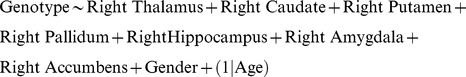
The starting model for the left hemisphere was:
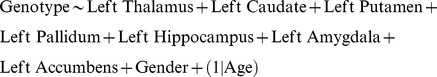
where “∼” means “modelled against”, “+” means inclusion of an explanatory variable in the model, and, “(1|Age)” means that Age is included as a random effect.

All explanatory variables (EVs) were assessed for collinearity. The volume of the right thalamus was found to be collinear with the volume of the right hippocampus, the volume of the right amygdala was also found to be collinear with the right pallidum, the left thalamus was found to be collinear with the left pallidum and the left amygdala was found to be collinear with the left hippocampus. Therefore, residual terms were used for these volumes, with the right thalamus regressed on the right hippocampus, the right amygdala regressed on the right pallidum, the left thalamus regressed on the left pallidum and the left amgydala regressed on the left hippocampus [59]. When two EVs are collinear, regression residuals of one variable relative to the other isolate the unique contribution of each explanatory variable independent from what is shared between them [60].

We fit the full right and left-side models as described above and then removed least significant terms from each model separately, checking for improved fit according to Akaike's Information Criterion (AIC) [61], [62], until a final model for each side was obtained [63]. AIC is a function of the likelihood, *L*, of the data given the model and the number of variables, in which better fitting models (i.e. those that match the observed data) have lower values, after a penalty has been applied for the number of explanatory variables included in the model. We have previously employed the AIC tool for successful model selection in an MRI and structural volume framework [60].

To determine if the final right or left hemisphere model was a better predictor of genotype, the fit between model and data for two final models was subsequently compared using the “anova” function in R [63].

## Results

### Demographic and Cognitive Characteristics

There were no significant differences between the groups in terms of any of the demographic or psychological measures taken (Table 1).

### Mixed-effect models for the left and right hemisphere to assess effect of genotype on bilateral grey matter structural volumes

The volumes of each deep grey matter structure segmented by FIRST were quantified in terms of both gross volume in mm^3^ (Table 2) and the volume normalised with total intracranial volume (Table 3). Normalised volumes were used for the development of all statistical models. Following model simplification, the optimal model for the right hemisphere included the right hippocampal volume and the right amygdalar volume (Table 4). Within the right hemisphere model however, only the right hippocampal volume was a significant main effect (p = 0.0136).

**Table 2:** Absolute volumes of deep grey matter structures in cubic millimetres for ApoE4+ and ApoE− groups.

	Neg		Pos		
	Mean	SD	Mean	SD	Diff.
Left Thalamus	8500	629	8465	782	−35
Right Thalamus	8121	670	8098	690	−23
Left Amygdala	1415	159	1431	207	16
Right Amygdala	1371	180	1467	217	96
Left Caudate	3966	487	4026	417	60
Right Caudate	4166	427	4207	424	41
Left Putamen	5379	528	5294	369	−85
Right Putamen	5383	544	5361	416	−22
Left Pallidum	1792	170	1797	113	5
Right Pallidum	1784	182	1828	128	44
Left Hippocampus	4231	403	4019	522	−212
Right Hippocampus	4296	317	3989	604	−307
Left Accumbens	664	126	650	125	−14
Right Accumbens	599	100	581	115	−18
Brain Stem Ventricle	22353	2480	23146	2988	793

Neg = ApoE4− group. Pos = ApoE4+ group. Diff = difference between Neg and Pos groups.

**Table 3:** Volumes of deep grey matter structures for ApoE4+ and ApoE4− groups with volumes normalised by total intracranial volume.

	Neg		Pos		
	Mean	SD	Mean	SD	Diff.
Left Thalamus	5.328	0.273	5.267	0.266	−0.061
Right Thalamus	5.090	0.298	5.042	0.272	−0.048
Left Amygdala	0.888	0.093	0.889	0.092	0.001
Right Amygdala	0.860	0.105	0.914	0.122	0.054
Left Caudate	2.487	0.293	2.513	0.272	0.026
Right Caudate	2.613	0.245	2.624	0.259	0.011
Left Putamen	3.368	0.226	3.303	0.224	−0.065
Right Putamen	3.371	0.244	3.342	0.215	−0.029
Left Pallidum	1.123	0.083	1.121	0.065	−0.002
Right Pallidum	1.117	0.076	1.139	0.064	0.022
Left Hippocampus	2.661	0.288	2.500	0.265	−0.161
Right Hippocampus	2.702	0.256	2.486	0.348	−0.216
Left Accumbens	0.416	0.075	0.405	0.074	−0.011
Right Accumbens	0.375	0.059	0.363	0.072	−0.013
Brain Stem Ventricle	14.010	1.360	14.400	1.470	0.390

Neg = ApoE4− group. Pos = ApoE4+ group. Diff. = difference between Neg and Pos groups. The following formula was used to compute normalized volumes of each deep grey matter structure:

total volume of GM structure (mm3)/total intracranial volume (mm3)×1000.

**Table 4:** Final, generalised mixed-effect model for genotype modelled against right hemisphere volumes.

	Estimate	Standard Error	z-value	p-value
Right Hippocampus	−4.155	1.684	−2.468	0.0136
Right Amygdala	7.449	3.924	1.898	0.0577

Formula: Genotype ∼ Right Hippocampus + Right Amygdala + (1|Age) where “∼” means modelled against, and “(1|age)” means that age is included as a random effect.

Fixed effects:

A generalised mixed-effect model is run using normalised volumes of right hemisphere grey matter structures and gender as explanatory variables together with age as a random effect. Genotype is set as the response variable. The final model is derived following an iterative model selection procedure that involves comparing successive models using Akaike's Information Criterion (see Methods for detailed description of model selection procedure).

The optimal model for the left hemisphere contained only the left hippocampal volume (Table 5).

**Table 5:** Final, generalised mixed-effect model for genotype modelled against left hemisphere volumes.

	Estimate	Standard Error	z-value	p-value
Left Hippocampus	−2.293	1.289	−1.779	0.0753

Formula: Genotype ∼ Left Hippocampus + (1|Age).

Fixed effects:

A generalised mixed-effect model is run using normalised volumes of left hemisphere grey matter structures and gender as explanatory variables together with age as a random effect. Genotype is set as the response variable. The final model is derived following an iterative model selection procedure that involves comparing successive models using Akaike's Information Criterion (see Methods for detailed description of model selection procedure).

A comparison of the left and right models indicated that the right hemisphere model explained the data significantly better than did the left hemisphere model (p = 0.01) (Table 6).

**Table 6:** Comparison of left and right hemisphere models.

	Df	AIC	BIC	logLik	p-value
Left Hem	3	63.202	68.555	−28.601	
Right Hem	4	58.582	65.719	−25.291	0.01008

Models:

Left hemisphere: Genotype ∼ Left Hippocampus + (1|Age).

Right hemisphere: Genotype ∼ Right Hippocampus + Right Amygdala + (1|Age).

The AIC value of the right hemisphere model is lower than that of the left hemisphere model. The right hemisphere model is also indicated to be a significantly better fit of the data than the left hemisphere model. See Methods for a detailed description of model comparison procedure and AIC calculation.

Abbreviations: Hem, Hemisphere; Df, Degrees of freedom; AIC, Akaike's Information Criterion Score; BIC, Bayesian Information Criterion; LogLik, Log-Likelihood.

### Regional Shape Change in the Left and Right Hippocampus

**Figure 2:**
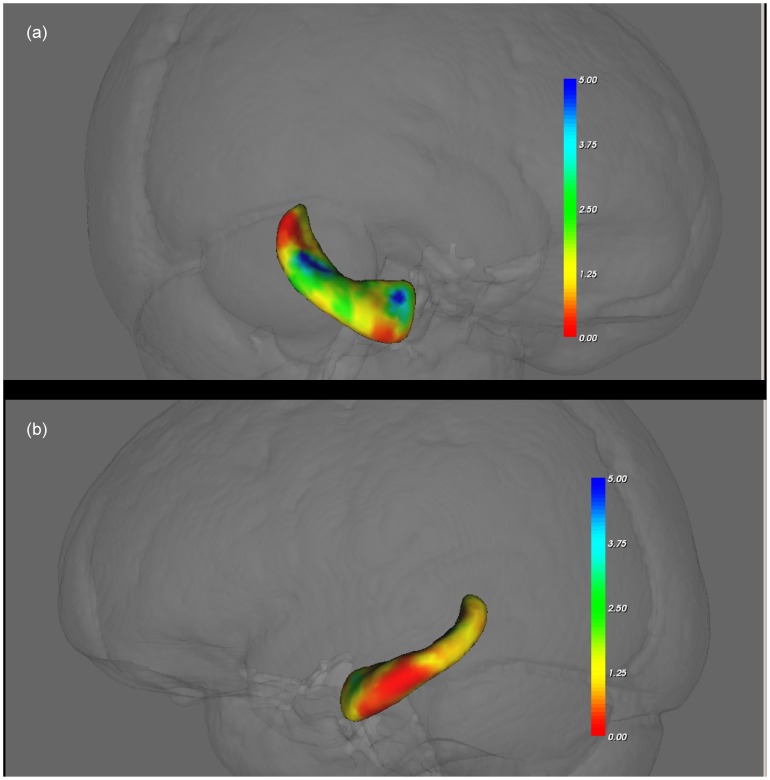
Regional shape changes in the left and right hippocampus using vertex analysis. Results show uncorrected F stats for the difference between ApoE4 carriers and non-carrier. The colour bar indicates the statistic values; an increase from red to blue is going from a lower to higher statistical significance. The right hippocampus is shown in the upper panel with the most significant differences between ApoE4 carriers and non-carriers shown in blue. The lower panel shows the left hippocampus. Note that there are no blue regions indicated on the lower panel, indicating that significant shape change is more pronounced in the right hippocampus.

Regional shape changes in the left and right hippocampus were assessed using vertex analysis within FIRST program. Vertex analysis creates a 3D mesh displaying the results of vertex analysis (Fig. 2). The uncorrected F stats are shown for the difference between ApoE4 carriers and non-carrier. The colour bars indicate the statistic values; an increase from red to blue represents progression from lower to higher statistical significance. In the right hippocampus (Fig. 2, upper panel), blue regions indicate the areas of most pronounced shape change between ApoE4 carriers and non-carriers. In the left hippocampus (Fig. 2, lower panel), there is little significant regional shape change between carriers and non-carriers. Vertex analysis which corrects for multiple comparisons however showed no significant region shape changes between carriers and non-carriers for either the left or the right hippocampus. This result is expanded upon in the discussion section.

## Discussion

The current results indicate that hippocampal volume is reduced in healthy young E4 carriers relative to non-carriers with the right hippocampus being more susceptible to atrophy than the left hippocampus. Analysis of regional shape changes also highlighted specific regions of the right hippocampus where ApoE4 carriers experienced atrophy relative to non-carriers. Such regional shape changes in ApoE4 carriers were absent in the left hippocampus. Overall, these results suggest that in ApoE4 carriers, the right hippocampus is directly vulnerable to atrophy in healthy young subjects.

Despite the negative effects of ApoE4 genotype on hippocampal volume, ApoE4 carriers still maintained an equivalent cognitive performance relative to non-carriers in a range of tests that probed verbal learning and memory, visual memory, working memory and attention. This suggests that although early atrophy may be occurring in ApoE4 carriers in a structure that is known to be affected in the early stages of AD, actual memory performance is not yet undermined by this atrophy.

The ApoE4 allele is the most well studied risk gene for AD, and previous work has found that the E4 allele is associated with increased atrophy of the hippocampus in Alzheimer's disease [64]–[67]. In healthy middle-aged and older non-demented E4 carriers, lower hippocampal volumes, decreased cortical thickness and increased rate of hippocampal atrophy relative to E4 non-carriers have been noted [20], [25], [35].

In healthy young subjects there are still relatively few studies which have examined the effect of ApoE genotype on brain structure and function. In the current study we hypothesised that hippocampal volume would be reduced in healthy young E4 carriers relative to non-carriers. The rationale for this hypothesis stems from earlier studies in younger populations, for example in young children and adolescents, E4 carriers were found to have thinner entorhinal cortices (EC) relative to E4 non-carriers [19]. Shaw et al. also showed a stepwise increase in cortical thickness in the EC, with E4 carriers having the thinnest cortex, E2 carriers having the thickest, and E3 homozygotes having an intermediate position. Similarly, in healthy young subjects (age ∼25 years) E3 homozygotes were found to have hippocampal volumes that were intermediate between E4 carriers who had the lowest hippocampal volume and E2 carriers who had the highest hippocampal volumes [68].

The results from the current study are in general agreement with these previous works. A generalised mixed-effect model for the right hemisphere indicated that genotype has an influence on right hippocampal volume and right amygdalar volume. However, only the right hippocampus was a significant fixed effect in this model. For the mixed-effect model of the left hemisphere, only the left hippocampus remained as a fixed effect following model simplification. Overall, the results from our mixed-effects models indicate that ApoE genotype has a significant effect on hippocampal volume. The volumes of no other structures were found to be significantly affected by genotype in the current study. Therefore our results extend the current literature by highlighting that the vulnerability of the E4 carriers to structural atrophy is localised to the right hippocampus while there is a general preservation of all other grey matter structures examined. One previous study has also noted that there were no differences in ventricular or hemisphere volumes between healthy young E4 carriers and non-carriers [69]. However, the current results provide more detailed confirmation of a preservation of deep grey matter structures outside of the hippocampus in healthy young E4 carriers. Together, these results support the concept that E4 status does not have a global effect on the brain regions, but rather leads to a selective targeting of the hippocampal structure.

There are some earlier studies which failed to find differences in hippocampal volume between healthy young E4 carriers and non-carriers [26], [70]. These discrepancies may stem partly from low sample sizes and partly from differences in the genotypes being studied. One previous study [26] examined differences between 10 E2/E3, 10 E3/E3 and 13 E3/E4 subjects and did not find hippocampal volume differences between these three groups, while the current study found hippocampal volume differences between a non-carrier group comprised of 22 E3/E3 subjects and a carrier group comprised of 21 E3/E3 subjects and one E4/E4 subject. The larger sample size of the current study, together with the automated algorithm for segmentation may enable more accurate detection of subtle volume changes between carriers and non-carriers. A second study which failed to find hippocampal volume differences between carrier and non-carrier groups [70] also employed manual segmentation and included a very heterogeneous group of carriers (4 E4/E4 subjects, 12 E3/E4 subjects and 2 E2/E4 subjects) and non-carriers (100 E3/E3 subjects, 2 E2/E2 subjects, 15 E2/E3 subjects). Additionally, the E2 allele variant has been reported to have a protective effect against AD [71] and cardiovascular diseases [72], and is also associated with increased longevity [73]. Thus it is preferable to exclude the E2/E4 genotype from the E4 carrier group. Future studies with larger cohorts should consider stratifying ApoE groups into more homogenous subgroups. Considering that the differences in hippocampal volume between these groups are subtle, more consistent stratification might help to clear up some of the discrepancies in the literature.

Volume changes within E4 carriers may be related to changes in synaptic connections and myelination of the peripheral cortical neuropil in E4 carriers [19], [68]. Within young ApoE4 targeted replacement (TR) mice also show lower spine density in cortical layers II/III compared to ApoE2 TR mice [74]. These differences may be related to increased oxidative insults resulting from changes in the pro-oxidant/antioxidant balance in E4 carriers [68], [75]. WM tract volume has also been shown to be reduced in healthy young E4 carriers [76]. These findings suggest that E4 status has a negative effect on both GM and WM structures in healthy young people. However, the absence of differences in memory performance between carriers and non-carriers in the current study and in earlier studies [68] [70], [77], suggests that the brain retains enough reserve capacity at a young age to avoid decline in cognitive performance despite the structural deficits outlined above in E4 carriers. Deficits associated with ApoE4 are more apparent later in life when E4 carriers are more vulnerable to the cortical thinning observed in aging [78] and AD [79], since less cortical thinning is necessary in key brain regions in E4 carriers before a critical anatomical threshold is passed, and neural dysfunctions become clinically evident.

Our finding of a more pronounced main effect of ApoE4 genotype on right hippocampal volume also extends the literature regarding laterality which has focused to date on older subjects where greater atrophy in the right hippocampus in E4 carriers has also been consistently reported [18], [37]–[39], [41], [80], [81] as well as among AD patients [8], [40], [66], [82]. Interestingly, in healthy controls a “normal” asymmetry appears to exist with the right hippocampus generally being larger than the left hippocampus; a finding which has been confirmed in a meta-analysis of 82 studies [36]. In older subjects, reversal of this typical asymmetry has been proposed as an indicator of early pathology [37], [39]–[41].

In ApoE4 non-carriers, our results show that mean normalised volume of the right hippocampus was marginally, though non-significantly, larger than mean left hippocampal volume. This finding is consistent with the usual asymmetry reported by the meta-analysis noted above [36]. Conversely, in ApoE4 carriers mean normalised volume of the right hippocampus was marginally, though non-significantly, *smaller* than left hippocampal volume. Thus, the current results point to a trend towards a reduction in the “normal” asymmetry of the hippocampus which has been noted in earlier studies in healthy older and AD cohorts [36], [37], [39]–[41]. Importantly, when comparing both the left and right hemisphere models, the right hemisphere model was also found to be a significantly better fit for the data, a finding which again emphasizes the selective vulnerability of the right hippocampus in ApoE4 carriers.

A greater predilection for damage in the right hemisphere has been noted in fMRI studies. Older E4 carriers have been found to exhibit more intense activation in parietal, frontal and right medial temporal lobe regions than non-carriers during the encoding of a picture learning task [13]. E4 carriers have also been found to show reduced activation in left hippocampal regions compared to E3 carriers, which also supports the model of greater compensatory changes occurring in the right hemisphere [13]. These studies are broadly compatible with the concept of greater right hemisphere involvement in normal aging as proposed by the Hemispheric Asymmetry Reduction in Older Adults (HAROLD) model of Cabeza [83].

Although not all fMRI studies have reported increased recruitment of right hemisphere activation in E4 carriers [84]–[87], differences between studies may be partly accounted for by the choice of functional tasks employed. A spatial context memory task which involves the right hemisphere in visuospatial processing was used in the study which found the greatest amount of compensatory right hemisphere activation [88]. The lack of a right hemisphere effect in other studies [84]–[87] may be related to tasks with an emphasis on language that would activate the left rather than the right hemisphere [33].

Findings of increased functional connectivity between medial temporal lobe (MTL) regions and other regions known to be affected by AD (e.g. posterior cingulate) in young E4 carriers also suggest that ApoE begins to be expressed in AD-associated brain regions long before cognitive decline [29]. Filbey et al. reported that young E4 carriers showed more medial frontal, cingulate and MTL activity compared to non-carriers in a working memory task [27]. In general agreement with this, other work has found that E4 carriers have more default mode network (DMN) connectivity and more hippocampal activation during a memory encoding task than non-carriers [28]. However, a study by Mondadori et al. [26] found that E4 carriers exhibited less neural activity in bilateral MTL and left frontal regions during the encoding and retrieval portions of an episodic memory task than performance-matched non-carriers. This was attributed to enhanced neural efficiency of memory networks in young adult E4 carriers which offers some support for a model of antagonistic pleiotrophy. Although hippocampal volume is reduced in the current cohort of healthy young E4 carriers, no cognitive differences were noted between carriers and non-carriers. Whether or not this equivalence of performance is achieved through extra compensation in the E4 carriers is not possible to say. It may be the case that cognitive deficits only become evident in E4 carriers when the risk allele is compounded by an additional risk factor such as AD history in the family [89].

In old age, the majority of studies note that E4 carriers have greater rates of cognitive decline compared with non-carriers [24], [90]. It could be hypothesized that structural changes occurring in healthy twenty year olds as a result of possession of the E4 allele, may not affect cognitive function at this early stage but may lay the ground work for faster cognitive decline in older age. Although there are exceptions, most studies have noted that E4 carriers performed worse in tasks of verbal and visual episodic memory compared with non-carriers. Also, studies have noted that those with two E4 alleles experienced more memory decline before those with only one E4 allele [91].

A limitation of the current study is that we do not know how the subjects progress over time. A longitudinal study which would follow healthy young carriers and non-carriers of the E4 allele over a period of ten or more years is warranted. Although there may be some limitations with regards to the FIRST algorithm, each subject's segmentations were carefully examined and found to be of good quality. The FIRST algorithm may offer some advantages over voxel-based morphometry (VBM) as VBM is prone to registration artefacts in deep GM structures [92]. FIRST is also more objective than manual segmentation methods which may not be sufficiently sensitive to detect subtle regional changes and localised volume loss. The algorithm proceeds with segmentation based on the intensity values of voxels and avoids the biases that arise when a researcher must visually judge contrasts in order to delineate boundaries during manual segmentation.

Overall, our results suggest that in the E4 carrier group, even among healthy subjects as young as 25 years of age, there are subtle structural changes in the hippocampus leading to volume reduction which are significant in the right hemisphere. Our results lend support to a growing body of evidence that indicates that the right hemisphere may have a greater predilection for damage in the very early stages of neurodegeneration. Our results also suggest that E4 carriers that exhibit volume reduction in the right hippocampus may be at greater risk of neurodgeneration in later life and that the structural deficits found in young carriers may not be clinically manifest until much later time points. However, future studies with larger sample sizes, as well as longitudinal studies will be needed to confirm this.
